# Program of neuropsychological stimulation of cognition in students: Emphasis on executive functions – development and evidence of content validity

**DOI:** 10.1590/1980-57642016dn11-010013

**Published:** 2017

**Authors:** Caroline de Oliveira Cardoso, Natália Martins Dias, Alessandra Gotuzo Seabra, Rochele Paz Fonseca

**Affiliations:** 1 Department of Psychology, Pontifícia Universidade Católica do Rio Grande do Sul, Porto Alegre, and Universidade Feevale, Novo Hamburgo, RS – Brazil.; 2 Department of Psychology, Centro Universitário Fundação Instituto de Ensino para Osasco, São Paulo SP – Brazil.; 3 Department of Psychology, Universidade Prespiteriana Mackenzie, São Paulo SP – Brazil. 4Department of Psychology, Pontifícia Universidade Católica do Rio Grande do Sul, Porto Alegre RS – Brazil.; 4 Department of Psychology, Pontifícia Universidade Católica do Rio Grande do Sul, Porto Alegre RS – Brazil.

**Keywords:** executive functions, neuropsychological intervention, cognitive stimulation, children

## Abstract

**Objective:**

The goal of this study was to describe the construction process and content validity evidence of an early and preventive intervention program for stimulating executive functions (EF) in Elementary School children within the school environment.

**Methods:**

The process has followed the recommended steps for creating neuropsychological instruments: internal phase of program organization, with literature search and analyses of available materials in the classroom; program construction; analysis by expert judges; data integration and program finalization. To determine the level of agreement among the judges, a Content Validity Index (CVI) was calculated.

**Results:**

Content validity was evidenced by the agreement among the experts with regards to the program, both in general and for each activity. All steps taken were deemed necessary because they contributed to the identification of positive aspects and possible flaws in the process.

**Conclusion:**

The steps also helped to adapt stimuli and improve program tasks and activities. Methodological procedures implemented in this study can be adopted by other researchers to create or adapt neuropsychological stimulation and rehabilitation programs. Furthermore, the methodological approach allows the reader to understand, in detail, the technical and scientific rigor adopted in devising this program.

## Abstract (pt)

**Objetivo:**

O objetivo desse estudo foi descrever o processo de construção e evidências de validade de conteúdo de um programa de intervenção precoce-preventiva para estimular as FE em crianças do Ensino Fundamental no ambiente escolar.

**Métodos:**

O processo seguiu as etapas recomendadas para construção de instrumentos neuropsicológicos, são elas: fase interna de organização do programa, com pesquisa bibliográfica e análise dos materiais disponíveis em sala de aula; construção do programa; análise de juízes especialistas; integração dos dados e finalização do programa. Para determinar o nível de concordância entre os juízes, foi calculado o Índice de Validade de Conteúdo.

**Resultados:**

Verificou-se concordância entre os especialistas tanto para o programa de forma geral, bem como em cada atividade, o que permitiu obter evidências de validade de conteúdo. Considera-se que todas as etapas foram essenciais e necessárias, pois contribuíram para identificar os pontos positivos e as possíveis falhas, assim como adequar os estímulos e aprimorar as tarefas e atividades do programa.

**Conclusão:**

Ao descrever os procedimentos metodológicos adotados nesse estudo, entende-se que outros também podem se basear para construir ou adaptar programas de estimulação e reabilitação neuropsicológica. Além disso, possibilita ao leitor uma compreensão detalhada de todo cuidado e rigor teórico e científico adotado na elaboração desse programa.

## INTRODUCTION

Neuropsychology, a segment of neuroscience, is applicable in different professional settings, such as clinical and educational. Many studies and systematic reviews seeking to structure a body of knowledge in the field of neuropsychological rehabilitation and provide a higher level of evidence have been conducted.^1-5^ In this context, actions and interventions primarily target patients with some kind of brain damage,^6^ with the main goal of remedying cognitive losses caused by this damage. These interventions constitute a tertiary level of prevention.

Yet, despite the primary interest in rehabilitating cognitive deficits, another type of intervention aimed at preventing or improving levels of health and well-being has been gaining prominence among researchers, clinicians and educators. Such interventions, through preventive and promotional actions, are destined to strengthen and improve cognitive and emotional processes among children with typical development.^7-9^ These interventions represent the incorporation of neuropsychology into the educational context.

Executive functions (EF) are among the cognitive functions that have been targeted by early and preventive intervention programs. They are a group of abilities that manage and regulate cognitive, emotional, and behavioral functions.^10-12^ These abilities include three main components: inhibitory control, cognitive flexibility, and working memory.^11^ A large number of studies have associated these abilities with better school performance and with social and emotional competencies in children.^13-17^ The same studies suggest that executive deficits can increase the risk of developing learning and behavioral issues, as well as psychopathologies.^18,19^ The relevance of executive functions justifies the emphasis that researchers have been giving to interventions that improve these abilities during childhood, at home and at school.

Current early and preventive interventions use numerous methods, including computerized cognitive training, non-computerized games, physical activities, mindfulness, school curricula, and extracurricular programs.^20^ Some programs have already been tested, such as the computerized program Cogmed,^21,22^ the extracurricular school program *Sarilhos do Amarelo,*^23^ and the curricula Tools of Mind.^24-26^ Despite the growing international emphasis on this type of intervention, national studies are still incipient. In Brazil, the Intervention Program for Self-Regulation and Executive Functions (*Programa de Intervenção em Autorregulação e Funções Executivas – PIAFEx*^8^) is noteworthy. It was developed, and has proven efficacy, to stimulate children at pre-school age^27^ and at first grade^28^ within the school setting.

Although studies provide information about the effectiveness of such programs, scant studies have systematically described the procedures used to develop intervention programs. Moreover, few studies report evidence on content validity. One possible source is to consult and follow the procedures and the theoretical, technical, and scientific rigor used in the development of standardized neuropsychological evaluation instruments.^29-32^ Fonseca et al.,^33^ for example, proposed a flowchart with each of the stages of the delicate adaptation process of neuropsychological instruments with verbal stimuli for use in Brazil. According to the authors, there are four essential stages:

[1] translation;
[2] analysis by non-expert judges;
[3] analysis by expert judges; and
[4] pilot study. Moreover, the authors of the instrument must analyze suggestions and adapt it based on suggestions given at each stage.

In this scenario of instrument development, it is essential to be cautious when dealing with content validity. Even though this investigation is closely tied to evaluation, it must also be applied to the development of intervention instruments. In this sense, if, in the context of evaluation, an appropriate battery must include items that explore a wide and representative range of the domain to be evaluated,^34-36^ then an intervention program must also be clear about the demands of each activity. Some strategies to verify evidence of content validity are the use of a consistent theoretical model to substantiate the development of activities, as well as subsequent analysis performed by expert judges.

Based on the previously mentioned stages for devising and adapting neuropsychological instruments, the objective of this study was to present the process of construction and evidence of content validity of the Program of Neuropsychological Stimulation of Cognition in Students: emphasis on Executive Functions, or PENcE (the acronym is from the original name in Portuguese, *Programa de Estimulação Neuropsicológica da Cognição em Escolares: ênfase nas Funções Executivas*).^37^ This program has an early and preventive characteristic, and is based on assumptions from neuropsychology and educational practices. The PENcE was planned to complement school curricula and targets school-aged children in 3^rd^ and 4^th^ grades of Elementary School. Through many cognitive and playful activities, as well as the teaching of strategy in a systematic and explicit way, the program stimulates and leverages EFs and correlated processes for subsequent use in other contexts.

## METHODS

**Figure 1:**
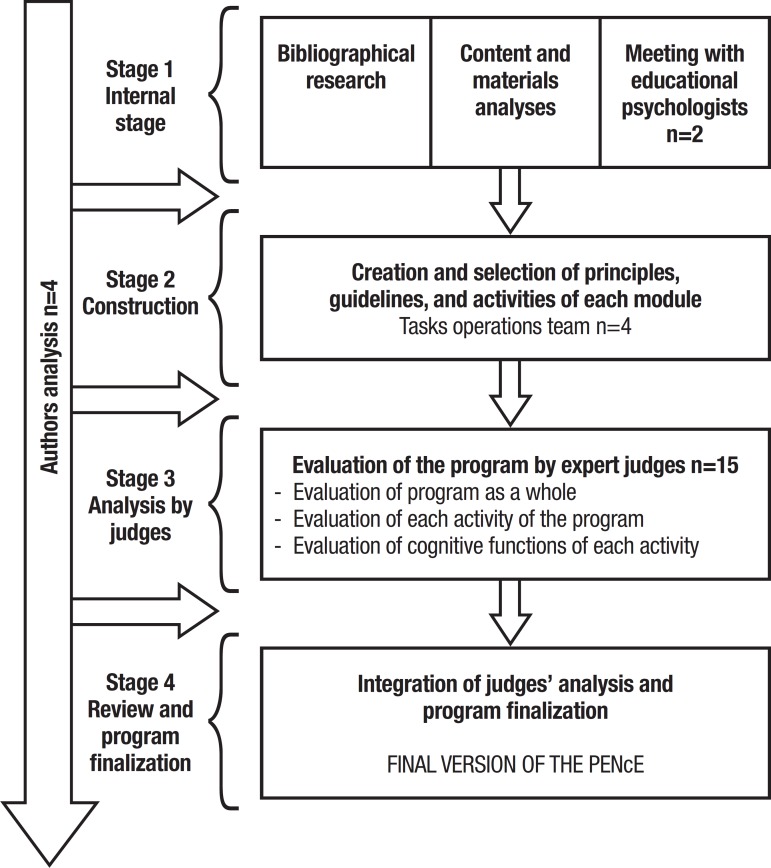
Flowchart depicting each stage of the PENcE construction process.

The process of creating the PENcE took place in four stages:

[1] Internal stage of program organization;
[2] Program construction;
[3] Analysis by expert judges; and
[4] Integration of judges' analysis and program finalization, as shown in the flowchart in Figure 1. The stage of analysis by expert judges was conducted with 15 professionals: 1 educational psychologist, 2 educators, 3 speech-language pathologists, and 9 psychologists with experience in neuropsychology. Each module of the program was evaluated by three expert judges. Three other judges assessed the program as a whole and analyzed which main executive component(s) is(are) involved in each activity. Table 1 shows area and level of education of each judge in each module.

**Table 1:** Education of expert judges.

Judge	Academic background	Level of education	Length of experience in neuropsychology
**Module 1: Organization and Planning **			
Judge 1	Educational Psychology	Expert in Neuropsychology	4 years
Judge 2	Education	PhD student in Developmental Disorders	5 years
Judge 3	Psychology	Master in Psychology	6 years
**Module 2: Inhibitory Control**			
Judge 1	Language and Linguistics	Master in Developmental Disorders	5 years
Judge 2	Psychology	PhD in Developmental Disorders	12 years
Judge 3	Speech-language Pathology	PhD in Psychology	7 years
**Module 3: Working Memory**			
Judge 1	Psychology	PhD in Psychiatry and Medical Psychology	12 years
Judge 2	Psychology	PhD in Psychology	11 years
Judge 3	Speech-language Pathology	PhD in Psychology	7 years
**Module 4: Cognitive Flexibility**			
Judge 1	Psychology	PhD in Psychology	10 years
Judge 2	Psychology	PhD Student in Psychology	10 years
Judge 3	Speech-language Pathology	PhD in Psychology	12 years
**Analysis of whole program**			
Judge 1	Psychology	PhD Student in Psychology	12 years
Judge 2	Psychology	PhD in Psychology	8 years
Judge 3	Psychology	Expert in Neuropsychology	5 years

**Procedures and instruments.** The present study was approved by the Research Ethics Committee of the Pontifical Catholic University of Rio Grande do Sul. In each stage, authors held brainstorming sessions and made the changes necessary to the study, creating new versions. Below, in Figure 1, each stage of the PENcE is presented and explained.

*Stage 1.* Internal stage of program organization: this stage was organized in three sub-stages:

[1] Literature review about EF intervention and stimulation programs among children;
[2] Analysis of didactic content during the first years of Elementary School. For the period of two months, one of the authors observed a 3^rd^ grade class with the goal of understanding classroom dynamics, materials used, content, and curricular matrices from the school;
[3] Meetings with two educators (teachers) with the objective of analyzing the possibility of including the program in the school curriculum, as well as obtaining more information about the school environment. Before creating the program, meetings were held with the team to think about the program's construction and to decide on next steps.

Stage 2. Program construction – The group established the frequency and length of meetings, as well as by whom the program would be mediated. General principles and guidelines to govern the program were created, and activities developed, adapted, and selected. The authors used the bibliographic review performed in Stage 1^38^, analysis of other programs^8,23,25,39,^, analysis of games known to the public (for example, Finders Keepers, Uno, and Mastermind), and neuropsychology paradigms (go no-go, cancellation tests). Other tasks were created specifically for the intervention. This stage also had a team to operationalize tasks comprising an educational psychologist and two psychology students. At the end, the authors conducted a new brainstorming session and activities were subsequently organized and systematized so that each would have instructions and a description.

*Stage 3.* Analysis by expert judges – This procedure was carried out in three sub-stages:

[1] Evaluation of the program as a whole: within and between sections, and organization of modules;
[2] Evaluation of each activity of the program;
[3] Evaluation of which function(s) or cognitive/neuropsychological component(s) is (are) stimulated in each activity. Also, using open-ended questions, judges were able to offer suggestions about changes or regarding new stimuli. For each module of the program, 3 expert judges gave their opinions and suggested adjustments. Judges answered an evaluation protocol (Figure 2 – example of the evaluation protocol used) in which each item of the activities was assessed using a Likert scale ranging from 1 to 4 points (1 – strongly disagree, item is not representative; 2 – disagree, item needs substantial reviews; 3 – agree, but item needs some reviews; and 4 – strongly agree, item is representative).

**Figure 2:** Example of evaluation form used in judges' analytical process.

	1	2	3	4	N/A
**Pre and post-module activity**					
Is it appropriate for children in 3^rd^ and 4^th^ grades of ES?					
Is it possible to be done in the classroom?					
Are descriptions of tasks and instructions clear?					
Is there coherence between activities and the proposed goal?					
**Stage 1: Psychoeducation**					
Is it appropriate for children in 3^rd ^ and 4^th^ grades of ES?					
Is it possible to be done in the classroom?					
Are descriptions of tasks and instructions clear?					
Is there coherence between activities and the proposed goal?					
**Stage 1: Modeling**					
Is it appropriate for children in 3^rd ^ and 4^th^ grades of ES?					
Is it possible to be done in the classroom?					
Are descriptions of tasks and instructions clear?					
Is there coherence between activities and the proposed goal?					

*Stage 4.* Integration of judges' analysis and program finalization – After the analysis of the judges, each activity was thoroughly evaluated and discussed among the team. There were some reformulations, as well as a detailed review. The latest version of the PENcE was then generated.

**Data analysis.** A Content Validity Index (CVI) was calculated to determine the level of agreement between judges. According to Alexandre and Coluci^40^, CVI allows measuring of the percentage of experts who agree with respect to a certain item, when Likert scales are involved. The index is calculated by adding the items rated 3 or 4 by the experts, and dividing the sum by the total number of responses in the item's evaluation. For this to be representative in groups of five or less subjects, everyone must agree. The following formula was used:

$$\mathrm{CVI}=\frac{\mathrm{Number}\;\mathrm{of}\;\mathrm{responses}\;"3"\;\mathrm{or}\;"4}{\mathrm{Total}\;\mathrm{number}\;\mathrm{of}\;\mathrm{responses}}$$

## RESULTS

Results will be presented for each stage of the PENcE.

**Results of Stage 1: Internal stage of program organization.** A systematic review^38^ was performed through bibliographical review and analysis of interventions already present in literature. In this review, 19 studies showed that EFs can be stimulated and developed in children with typical development. Furthermore, there was a predominance of studies using computerized programs, which involved, for the most part, stimulating working memory. Other studies used tasks involving pencil and paper, and some programs were incorporated into the school curriculum with the objective of improving self-regulation. Depending on the program format, effects and gains vary. Methods, modes, and techniques used in these studies were an inspiration to create the PENcE. Curricular standards from the Department of Education, curricular matrix analyses, and observations of the school environment during 2 months, allowed a better understanding of this setting and of the contents and materials used in Elementary School.

**Results of Stage 2: Program Construction.** The program was organized in 4 modules, considering the main executive abilities: Module 1 – Organization and Planning; Module 2 – Inhibitory Control; Module 3 – Working Memory; and Module 4 – Cognitive Flexibility. Each module had the three following stages:

1. *Strategy acquisition:* Psychoeducation and Modeling – students are taught about what, where, how, and why use the strategies in each component of the EFs.
2. *Learning and Strategy Consolidation:* Students are stimulated to actively practice the strategies that were taught through playful and cognitive activities, as well as school-related tasks. For each module of the PENcE, this stage initially included from 6 to 8 activities, all developed based on information acquired in Stage 1.
3. *Reflection and Transfer to School and Everyday Activities:* Students reflect about how learning and strategies can be applied to various aspects of life and to school activities.

The program was outlined to be developed within the school environment, 3 times a week in 50-minute sessions, for 5 months. These sessions were mediated by the teacher, and assisted by a neuropsychologist. A story was created based on the movie "A Bug's Life." In this plot, each module was represented by an "Ant" that, facing its challenges, was helped by the "Active Mind Ants League." The "Ants," along with the "Active Mind Ants League," present the strategies to the students, encouraging them to learn and engage in the activities. For each module, activities were organized and included descriptions of tasks, instructions, and materials used. At the end of each module, a section called "Reviewing the Modules" was added, so that the abilities stimulated in the previous modules could be reviewed and integrated. At the end of this process, the first version of the program was reviewed by the authors and submitted to the evaluation by expert judges.

**Results of Stage 3: Analysis by Judges.** Tables numbered from 2 to 9 show the evaluation of judges after the first version of the program. The tables were organized by modules, with their respective activities.

The overall evaluation of the program (Table 2), as well as the evaluation of each module (Table 3), showed a level of agreement (CVI) of 1. This suggests that, from the judges' perspective, each module stimulates what it intends to. Also, sequence and module organization are both adequate, as were activity length and order. With respect to specific modules (Tables 5 to 9 - available on the site), there was also agreement among judges on the vast majority of activities, showing that: there is coherence between the activity and its goal; activity descriptions are clear; and activities are adequate for the target age group and can be implemented in the school setting.

**Table 2:** Results of global analysis of all modules by expert judges.

Evaluation criteria	Global Analysis Module 1				Global Analysis Module 2				Global Analysis Module 3				Global Analysis Module 4			
	J1	J2	J3	CVI	J1	J2	J3	CVI	J1	J2	J3	CVI	J1	J2	J3	CVI
Does the module as a whole stimulate what it proposes to?	4	4	4	**1**	4	4	4	**1**	4	4	4	**1**	4	4	4	**1**
Is the order of activities adequate?	4	4	4	**1**	4	4	4	**1**	4	4	4	**1**	4	3	4	**1**
Are the suggestions on how to adapt this knowledge into school activities adequate?	3	3	4	**1**	3	3	4	**1**	4	4	4	**1**	3	4	4	**1**

J1: Judge 1; J2: Judge 2;J3: Judge 3.

**Table 3:** Results of program analysis as a whole by expert judges.

Evaluation criteria	J1	J2	J3	CVI
Is the sequence of the 4 modules adequate?	4	4	4	1
Is the duration of each model adequate?	4	4	4	1
Are the descriptions and organization of the 3 stages comprising each of the modules coherent?	4	4	4	1
Is there coherence in the order of the activities within sections?	4	4	4	1
Is there coherence in the order of the activities between sections?	4	4	4	1

J1: Judge 1; J2: Judge 2;J3: Judge 3.

Table 4 shows which executive component(s) is (are) predominantly stimulated in each of the PENcE activities. In most activities, there was 100% agreement among judges. In the activities aimed at reviewing each module, the intent was to stimulate more than one component at the same time. Consequently, there was more than one percentage number. Activities that did not obtain a CVI equal to one on any of the evaluation criteria, or that did not have 100% agreement among judges with respect to the executive component that was stimulated, were reviewed and adapted. The revisions that were made are described in the section "Results of Stage 4: Integration of Judges' Analysis and Program Finalization."

**Table 4:** Analysis of demands/content of PENcE activities (executive predominant component in each activity).

Activity	Responses of Judges						Activity	Responses of Judges					
	Abil	P/O	IC	WM	CF	%		Abil	P/O	IC	WM	CF	%
Fitting Numbers	P/O	2	1			66.6	Creating an Object: Loose Lips Sink Ships*	P/O IC	3	3			100 100
Packing the Backpack for School	P/O	3				100	Where are the animals?	WM			3		100
Creating a Cover for the Notebook	P/O	3				100	Numbering the Sequence	WM			3		100
Dots Game	P/O	3				100	Organizing the Sequences	WM			3		100
Looking for the Diamond	P/O	3				100	Completing the Sentence	WM			3		100
Logical Sequence	P/O	3				100	There's One Missing	WM			3		100
Creating an Insect	P/O	3				100	Differences Game	WM	1		2		66.6
Cooking	P/O	3				100	Following Instructions*	WM IC		3	3		100 100
Writing an essay*	P/O	3				100	Crazy Sentences*	WM P/O	2		3		100 66.6
For Every Sound There's a Movement	IC	3				100	How Can We Solve This?	CF				3	100
Opposites Game	IC	3				100	A New Ending	CF				3	100
Looking for the Bullseye	IC	3				100	Looking Through Another Perspective	CF				3	100
Dancing	IC	3				100	Switching	CF				3	100
Controlling the Urge	IC	3				100	Combining Cards	CF				3	100
Simon Says	IC	3				100	A New Ending for "The Story of the Three Little Pigs"*	P/O IC CF	3	2		3	100 66.6 100
Card Game	IC	3				100	Building a Different Tower*	P/O FL	3			3	100 100
Not everything is what it seems**	IC			2	1	66.6	Picnic *	P/O FL	3			2	100 66.6
Birthday Party *	P/O IC	3	3			100 100							

Some activities have more than one level of complexity. For these activities, judges also evaluated whether the levels were equivalent, or level 2 was harder than level 1, or vice-versa. For all activities involving more than one level, the judges agreed that the level initially developed as the most complex one was, in fact, harder than the previous level. The judges suggested a few changes for some of the activities with the intent of making them clearer and easier to understand. Most of those changes were incorporated into the program. Some judges asked for more details or to add more information in the instructions, others proposed substituting the wording of some terms. After these considerations, the evaluation of judges was integrated into the latest version of the PENcE.

**Results of Stage 4: Integration of Judges' Analysis and Program Finalization.** After the judges' analysis, adjustment and changes were incorporated into each module:

Module 1 - Activity of Fitting Numbers: one of the judges understood that the activity stimulated, predominantly, inhibitory control. However, this judge also considered that the activity demanded planning and organization. To make the activity clearer, the following procedures were carried out: addition of instructions in writing, and directions for teacher to count the number of mistakes and pages that each student needed to complete the task. This helped check which students were able to plan the activity well before executing it.

Activity 5: Cooking – one of the judges understood that this activity was not suitable for the classroom setting due to time constraints. Therefore, the activity was broken down to be performed over two days. 1^st^ day: planning what is going to be cooked; 2^nd^ day: execution of activity and evaluation.

Module 2 - Activity 1: Dancing – some examples were added, upon request by one of the judges.

Activity 6: Not everything is what it seems – because of the understanding that the activity was not coherent and its description not clear, it was excluded from the program.

Module 3 - Activity 2: Numbering the sequences – rather than numbering a sequence using a material similar to a mat, it was decided that the stimuli would be projected onto a screen.

Activity 5: Differences Game – besides finding the differences between the images, students had to, at the same time, count from 50 to 20. Only after this countdown could they write down on a piece of paper the differences that they had observed.

Module 4 - Although the CVI was appropriate for all criteria, in activity 4, "Matching Cards," one rule was added: students had to combine the cards with images of animals. In addition to the colors of the animals and the habitat where they live, the activity included the number of syllables of each word, with the objective of making the task more complex and challenging.

When there were disagreements with respect to changes and adaptations, they were submitted to one of the authors, who established a consensus. There was then a review of the whole program, finalizing it. The summarized final version is given in the appendix of this study. In the PENcE book^40^, the whole program and its activities are described in detail so they can be replicated. After reading this material, other teachers can conduct the activities.

## DISCUSSION

This study presented the development process and content validity evidence of the PENcE for school-aged children. The PENcE is a program for stimulating EFs that, through various cognitive and school-related activities, as well as through the teaching of strategies, aims to systematically leverage these abilities in the school setting. Creating a rehabilitation or a neuropsychological stimulation program requires rigorous and well defined stages and procedures. However, there is still no systematization of the process in the available literature. Existing programs do not specify how they were devised or constructed and do not report content validity evidence. In this scenario, the present study sought to follow the same methodological and technical rigor used to adapt or create neuropsychological evaluation instruments^29,30,31,32,33^.

Pasquali^34^ proposed a model with the steps necessary to develop a psychological instrument, which must follow a sequence. These steps comprise three main axes: theoretical procedures (involving construct definition, producing each item of the instrument, and content validity); empirical procedures (planning and application of the instrument, data collection); and analytical procedures (statistical analyses verifying that the instrument is valid, reliable, and regulated). In this study, the first axis was attained, providing a direction for the ensuing stages. This was the first stage of the validity work and of the search for effectiveness of the program. All stages contributed to identify positive aspects, possible flaws, verify understandability and level of complexity, adapt stimuli, and improve tasks of the program.

The first stage included a long process of research, reflections on the theme, and discussions among the team. This required the exploration of national and international bibliographies. The school environment and curricular matrices of Elementary School were closely monitored. This was a fundamental stage because it allowed the definition of aspects and theoretical models that would become the basis of the PENcE, as well as the constructs that would be stimulated. Based on the model created by Diamond^11^, it was decided to include the central abilities (inhibitory control, working memory, and cognitive flexibility), which form the basis of the emergence of more complex or superior functions, and one complex executive function (planning and organization) because of its relevance to school performance. These definitions, along with a deeper understanding of other available studies, allowed the following steps to be developed underpinned by a solid theoretical basis.

In the construction stage, format, principles, and program guidelines were planned. Tasks that would compose each module, with their respective instructions and descriptions, were also planned. As is the case for the development of any rehabilitation program, some decisions had to be made, considering the objective of the program. Initially, it was decided that the program would be incorporated into the school curriculum. Programs of curricular adaptations adopt a wider and more naturalistic^41^ approach, and there is evidence that such programs tend to present better results in terms of acquiring abilities and transferring gains.^7,20,24,26,28,42,43^ It was also decided that the program would be based on the teaching of systematic and explicit strategies of the EFs. Meltzer^39^ holds that classroom interventions must include direct instructions of metacognitive strategies, and that they must be structured and systematic. Thus, with the goal of allowing more reflection and knowledge transfer to other contexts, numerous playful, cognitive, and school-related activities were proposed.

In the third stage, the judges' analysis revealed a high level of agreement among experts, both for the program as a whole and each activity. In this stage, the relevance of the tasks within the program was verified, allowing tasks to be adjusted and improved. The Expert Committee has the role of consolidating the presented version and helping produce the pre-final version of the instrument^31,44^. Also, agreement among experts provides confirmation of content validity ^34,36^. In the absence of other studies investigating evidence of content validity in cognitive stimulation interventions among healthy children, the index found here is in consistent with those of other studies determining this type of validity for instruments for neuropsychological evaluation^43^. The existence of evidence of validity with respect to content makes it easier for its applicability to be tested and checked in typical samples and different clinical groups. These procedures allowed the derivation of content validity evidence for the PENcE. After checking all suggestions put forward by the judges, in the fourth stage of the study, changes and adjustments were made, resulting in the final version of the intervention program.

Given the lack of research about the development process of rehabilitation or neuropsychological stimulation programs, this study displays an innovative approach. It can help other researchers and clinicians to develop strategies and intervention programs, since it provides a systematization and outline of the process. This study also provides readers with a detailed understanding about each stage and offers insight on all the care and rigor applied in producing the PENcE. The PENcE can be a useful tool to education and healthcare professionals, since it can provide orientation for their practices and promote benefits for school-aged children in the promotion of EFs. Future studies should verify the effectiveness of the program for children in Elementary School I, and adapt the program for use in teenagers and clinical groups with potential executive dysfunctions, such as attention deficit hyperactivity disorder (ADHD), and learning related disorders.

## APPENDIX

**Table:** Final version of the PENcE

**INTRODUCTION**
Program presentation and screening of "A Bug's Life."
**MODULE 1: ORGANIZATION AND PLANNING**
Strategy: three stages: planning (thinking it through before starting a task); execution (thinking during the performance of the task); and evaluation (reflect and evaluate whether what was planned was actually accomplished).
**Stage 1: Strategy acquisition stage: Psychoeducation and Modeling** Pre-module activity: Fitting Numbers Psychoeducation: introduction of "Beatriz, the Ant." She is a ballerina ant who has difficulties whenever planning something, getting confused when there are many things to do. Modeling: Packing the Backpack for School, and Creating a Cover for the Notebook.
**Stage 2: Learning and strategy consolidation stage ** The following activities are part of this module: - Dots Game; - Looking for the Diamond; - Logical Sequence; - Creating an Insect; - Cooking;- School-related Activities.
**Stage 3: Reflections and transfer to everyday and school-related activities** Post-module activity: Fitting Numbers Moment of reviewing module and strategies. Space for reflection and discussion.
**REVIEWING THE MODULES**
Writing an essay.
**MODULE 2: INHIBITORY CONTROL**
In order to stimulate this ability, the following strategy will be taught to the student: "Stop, Think, and Go On" (STG).
**Stage 1: Strategy acquisition stage: Psychoeducation and Modeling** Pre-module activity: For Every Sound There's a Movement. Psychoeducation: Introduction of "Pedro, the Ant." He really enjoys playing soccer, but he is very impulsive and has difficulties waiting for his turn. Modeling: Opposites Game, and Looking for the Bullseye.
**Stage 2: Learning and strategy consolidation stage ** The following activities are part of this module: - Dancing; - Looking for the Bullseye; - Controlling the Urge; - Simon Says; - Card Game; - School-related Activities.
**Stage 3: Reflections and transfer to everyday and school-related activities** Post-module Activity: For Every Sound There's a Movement Moment of reviewing module and strategies. Space for reflection and discussion.
**REVIEWING THE MODULES**
- Birthday Party; - Creating an Object: Loose Lips Sink Ships.
**MODULE 3: WORKING MEMORY**
Strategy - 4 stages are suggested: (1) pay attention to the stimuli/instructions; (2) memorize new information - repeating and mentally visualizing what is said; (3) mentally manipulate and organize the stimuli; (4) execute the activities slowly, focusing on quality.
**Stage 1: Strategy acquisition stage: Psychoeducation and Modeling** Pre-module activity: Where are the animals? Psychoeducation: Introduction of "Patricia, the Ant." She is an ant who likes fashion and would like to be a model. However, she usually forgets things and doesn't remember much of the information and instructions that are given to her. Modeling: Image Sequence: Body Parts, and Numbering the Sequence.
**Stage 2: Learning and strategy consolidation stage ** The following activities are part of this module: - Organizing the Sequences; - Differences Game; - There's One Missing; - Completing the Sentence; - Numbering the Sequence;- School-related Activities.
**Stage 3: Reflections and transfer to everyday and school-related activities** Post-module activity: Where are the animals? Moment of reviewing module and strategies. Space for reflection and discussion.
**REVIEWING THE MODULES**
- Following Instructions; - Crazy Sentences
**MODULE 4: COGNITIVE FLEXIBILITY**
Strategy: When something unexpected happens, or when trying to solve a problem, first, it is necessary to create various alternatives.
**Stage 1: Strategy acquisition stage: Psychoeducation and Modeling** Pre-module Activity: How Can We Solve This? Psychoeducation: introduction of "Fabio, the Ant." Fabio is the singer of his school band. When he faces a new or unplanned situation, he has difficulties thinking of different ways of solving the issue. Modeling: A New Ending to the Movie, and Collective Drawing.
**Stage 2: Learning and strategy consolidation stage ** The following activities are part of this module: - Looking From Another Perspective; - Switching; - Combining Cards; - Find Out the Code; - A New Ending;- School-related Activities.
**Stage 3: Reflections and transfer to everyday and school-related activities** Post-module Activity: How Can We Solve This? Moment of reviewing module and strategies. Space for reflection and discussion.
**REVIEWING THE MODULES**
Finalization of all modules. Activities: - A New Ending for "The Story of the Three Little Pigs"; - Building a Different Tower; - Picnic.
